# An Efficient Transformer Detector for Traffic Scenes via Lightweight Backbone and Multi-Scale Attention

**DOI:** 10.3390/s26196043

**Published:** 2026-09-24

**Authors:** Jinfeng Zheng, Yuan Zhang, Yanbo Hui, Xiaoxuan He, Xiaohui Zhang, Haiyang Ding, Qiao Wang, Bo Chen, Zongpu Nan

**Affiliations:** 1 School of Mechanical and Electrical Engineering, Henan University of Technology, Zhengzhou 450001, China; zhengjf@haut.edu.cn (J.Z.); yanbohui987@126.com (Y.H.); 2 College of Electrical Engineering, Henan University of Technology, Zhengzhou 450001, China; hexiaoxuan0513@163.com (X.H.); zhangxh@haut.edu.cn (X.Z.); wqiao2010@haut.edu.cn (Q.W.); chenbo@stu.haut.edu.cn (B.C.); n411327@163.com (Z.N.); 3 Weihua Group Co., Ltd., Changyuan 450001, China; 15821642742@139.com

**Keywords:** traffic target detection, attention mechanism, lightweight, multi-scale feature fusion

## Abstract

As autonomous driving systems shift from experimental testing environments to real-world open-road deployment, object detection in traffic scenes has emerged as a critical challenge in the perception pipeline. In complex traffic scenarios, significant variations in object scales (e.g., small vehicles and large infrastructure) and dynamic backgrounds often result in frequent missed detections and false positives under real-time constraints, which directly impairs driving safety. To tackle these issues, we propose Trans-DETR, an efficient end-to-end detector for traffic scenes based on a multi-scale attention mechanism and a lightweight backbone network. The conventional Convolutional Block Attention Module (CBAM) captures features at only a single scale, making it insufficient for handling multi-scale characteristics. To overcome this limitation, we introduce the Multi-Scale Attention Module (MSAM), which incorporates Multi-Scale Convolution Attention (MSCA) into the cross-scale fusion path of the model. This enables the effective extraction of multi-scale feature information and significantly improves detection accuracy. Furthermore, we propose the lightweight backbone network LiteSNet to reduce the parameter inflation and computational burden caused by traditional large-kernel convolutions. LiteSNet decouples large kernels into sequentially stacked horizontal and vertical separable convolutions, while employing dilated convolutions to expand the receptive field without information loss. This strategy significantly reduces both the parameter count and computational complexity while preserving the same effective receptive field as large kernels. Finally, we design a reparameterized detection head called RepHead, which deeply integrates cross-stage partial connections with reparameterization techniques. This allows for efficient fusion of low-level spatial details and high-level semantic features, thereby enhancing the model’s ability to detect small-scale traffic targets. Experimental results demonstrate that the proposed method achieves a mean average precision (mAP) of 89.8% on the KITTI dataset, outperforming RT-DETR-L by 2.0%. It also reduces the model size by 57% and computational complexity by 55.7%, with a single-frame inference time of only 4.6 ms. Compared with state-of-the-art approaches, the proposed algorithm achieves superior performance in terms of accuracy, efficiency, and model compactness, providing strong technical support for the advancement of autonomous driving systems.

## 1. Introduction

The deep integration of artificial intelligence and transportation systems is pushing driverless technology from closed test fields onto open roads [1,2]. Within the perception chain of autonomous driving, object detection in traffic scenes is expected to identify and locate vehicles, pedestrians, traffic signs and other targets accurately and in real time, supplying centimeter-level input for path planning, behavior prediction, collision avoidance and vehicle-road-cloud collaboration [3,4]. Since perception errors propagate directly into driving decisions, detection accuracy and latency are safety-critical rather than purely technical indicators, and both must be met under the tight computation and power budgets of onboard platforms [5].

Meeting these requirements remains difficult in complex traffic scenes. Object scales vary over a wide range: a nearby vehicle may cover thousands of pixels, while a pedestrian or cyclist far ahead often occupies fewer than 32×32 pixels, so the corresponding feature responses are sparse and easily missed [6,7], and background clutter, uneven illumination and mutual occlusion at intersections further aggravate the problem. Small and distant objects are also the most critical ones for driving safety, because they require the earliest response from the vehicle. At highway speeds, even a single missed detection of a distant pedestrian can shrink the time available for braking and evasive maneuvers to a level that threatens driving safety. However, small-object detection remains the weakest aspect of current detectors, and the accuracy gap between small and large objects is still substantial for mainstream models. Part of the reason is that commonly used attention mechanisms, such as CBAM [8], recalibrate features at a single scale and cannot adapt to such large scale differences. A further obstacle arises when such detectors are deployed: traffic perception has to run in real time on onboard hardware with limited computation and power [9,10], but accurate models are usually expensive, and RT-DETR-L alone requires 125.1 MB of parameters and 108.0 GFLOPs. Existing lightweight backbones reduce this cost only to a limited extent, since HGNet-v2 still carries structural redundancy and the large-kernel depthwise convolutions in efficient networks such as StarNet [11] suffer from computational costs that grow quadratically with kernel size. Meanwhile, the fusion strategy of the detection head also restricts the detection of small and occluded targets: shallow-layer spatial details that these targets rely on tend to be diluted during top-down aggregation because low-level and high-level features are not balanced in weight [12,13], whereas strengthening the fusion path generally introduces inference overhead that conflicts with the real-time requirement. Existing methods therefore tend to improve one of these aspects at the expense of the others. This trade-off reflects the inherent tension between model lightweightness and detection accuracy, because compressing representational capacity to satisfy real-time constraints generally weakens the localization of small and occluded objects. Few have addressed scale-robust feature extraction, efficient computation and detail-preserving fusion within a unified framework that reconciles rather than trades off these objectives.

Motivated by these observations, this paper proposes Trans-DETR, an efficient Transformer-based detector built upon RT-DETR. The proposed detector incorporates a Multi-Scale Attention Module (MSAM) to strengthen scale-robust feature extraction, a lightweight LiteSNet backbone to eliminate structural redundancy, and a reparameterized detection head (RepHead) to preserve shallow spatial details during feature fusion. The main contributions of this paper are summarized as follows:

1. To cope with large scale variation and the frequent missed detections of small objects, a multi-scale attention module (MSAM) built on multi-scale convolution attention (MSCA) is introduced and embedded into the cross-scale fusion pathway of the RT-DETR encoder, which improves the encoder’s ability to extract multi-scale traffic features without adding inference latency.
2. To reduce the quadratic computational growth caused by large depthwise kernels, a lightweight backbone termed LiteSNet is proposed, which decomposes the 7 × 7 depthwise kernels in StarNet into cascaded 1 × k and k × 1 separable convolutions with dilated aggregation, thereby cutting parameters and computation with little performance loss.
3. To relieve the loss of shallow spatial details caused by unbalanced feature weights in pyramid fusion, a reparameterized detection head named RepHead is designed by combining cross-stage partial connections with reparameterization, enabling efficient fusion of low-level details and high-level semantics and improving the detection of small-scale traffic targets.
4. Extensive experiments are conducted on the KITTI, PASCAL VOC2012 and BDD100K benchmarks, and the results demonstrate that the proposed Trans-DETR achieves a favorable balance among detection accuracy, computational efficiency and model compactness, confirming its practical value for real-time traffic scene perception.

The remainder of this paper is organized as follows: Section 2 provides an overview of the current developments in deep learning-based object detection algorithms and their applications in the transportation domain. Section 3 presents the network architecture of Trans-DETR. Section 4 details the experimental setup and performance analysis. Section 5 concludes by summarizing the contributions and limitations of this work.

## 2. Related Work

### 2.1. Deep Learning-Based Object Detection Algorithms

Object detection algorithms based on deep learning can be classified into two major categories. One is the two-stage object detection algorithms based on region proposals [14]. These algorithms first use region proposal methods to identify candidate regions that may contain objects from the input image, and then extract features and classify these candidate regions through convolutional neural networks [15]. Two-stage algorithms usually have higher detection accuracy, but they face a performance bottleneck between real-time performance and detection accuracy when dealing with complex and computationally intensive tasks. The other category is the one-stage object detection algorithms based on regression analysis, represented by the YOLO (You Only Look Once) series and the SSD (Single Shot MultiBox Detector) series. The YOLO series algorithms [16,17] have been widely applied in the field of computer vision, extracting features from the input image in one end-to-end network and then predicting the possible positions and categories of objects in the input image based on these features. SSD [18] combines the regression idea of YOLO and the anchor mechanism of Faster R-CNN, achieving efficient and accurate object detection through dense sampling on feature maps of different scales. One-stage algorithms achieve faster detection speeds than two-stage algorithms, but at the expense of detection accuracy [19]. Therefore, in object detection tasks in traffic scenarios with high real-time requirements, one-stage algorithms are often superior to two-stage algorithms.

### 2.2. Object Detection Algorithms for Traffic Scenarios

To address the challenges of large scale variations and high false detection rates of traffic signs in complex environments, Han [20] proposed the EDN-YOLO framework based on YOLOv5s. By integrating an EfficientVit lightweight backbone, a decoupled detection head, and a CIoU+NWD hybrid loss function, this approach significantly enhances global feature representation and improves the sensitivity to small objects without increasing computational cost. Wang [21] tackled the issue of reduced detection robustness caused by snow occlusion by reconstructing the C3Ghost module within the YOLOv5s architecture to reduce redundant computation and introduced the CA attention mechanism to explicitly encode spatial information, thereby enhancing the perception of small objects. Zhang [7] proposed NOC-YOLO to address the problem of texture deficiency and unclear boundaries of small vehicles in drone-captured infrared images. By introducing the attention mechanism and multi-scale feature fusion into YOLOv10n, the feature interaction and the representation ability of weak textures have been significantly enhanced, thereby achieving high-precision vehicle detection in night scenes. Yu [22] constructed the visible-infrared dual-mode MiVehicle dataset and designed the lightweight rotated bounding box detector ML-Det, which enhances feature expression and detection robustness for vehicles in arbitrary orientations without increasing model parameters. Wang [12] proposed CW-DETR, an RT-DETR-based detection transformer that tackles severe feature degradation in adverse weather by integrating a multipath feature enhancement network, a multiscale edge enhancement module, and an adaptive bidirectional feature pyramid, demonstrating strong robustness for traffic sign detection under rain, fog, and snow conditions. Yu [23] also proposed the Anchor-Free domain adaptation framework DAAF for urban environments. This framework achieves image-level global alignment through fully convolutional adversarial training with texture constraints, and then employs a pixel-level strategy for local feature transfer. Wang [24] introduced the lightweight detection framework MF-YOLO, which utilizes a convolution-ViT hybrid backbone to capture both local details and global context. The lightweight FPN neck incorporates an attention mechanism to focus on critical features, enabling high-precision detection of small objects while maintaining compatibility with mobile deployment. Zhang [13] proposed BANet, an efficient lane detection network that leverages bidirectional feature aggregation to hierarchically integrate complementary cross-scale cues and adaptive multi-scene perception to dynamically enhance lane-relevant features under diverse background conditions, achieving state-of-the-art performance on CULane, CurveLanes, and TuSimple benchmarks. Liu [25] proposed a cascaded recognition framework tailored for foggy conditions, integrating the pix2pixHD defogging network with an improved YOLOv5-Transformer architecture to effectively reduce false detections caused by fog interference. Recent studies have further explored multi-scale feature modeling and attention mechanisms for driving perception. Zhang [26] proposed MRGD-Net for driver attention prediction, in which multi-scale features are reconstructed and a gated dual-attention module is designed to adaptively emphasize salient regions while suppressing redundant information, verifying the effectiveness of combining multi-scale modeling with gated attention in complex driving scenes. Cui [27] developed TS-DETR on the basis of RT-DETR, where a multi-sequence feature fusion strategy together with enhanced channel and spatial attention is incorporated to acquire more comprehensive multi-scale information for traffic sign detection and recognition. Shi [28] proposed LDA-DETR, a lightweight dynamic attention-enhanced detector that improves small object detection while reducing model complexity. Liu [29] introduced WDFS-DETR, which integrates wavelet-based attention with hierarchical multi-scale feature alignment to achieve efficient small object detection on embedded platforms. Despite significant advancements made by existing traffic object detection algorithms in processing traffic scenes, numerous challenges persist in areas such as detection accuracy, model complexity, and inference efficiency. These challenges hinder their capacity to offer dependable support in autonomous driving and intelligent transportation systems.

## 3. Methodology

RT-DETR first extracts multi-scale high-level features from the input image through a backbone network and selects feature maps from the last three stages as the encoder’s input [30]. The encoder consists of two key components, the intra-scale interaction and fusion encoder (AIFI) and the cross-scale feature fusion module (CCFM), which jointly encode multi-scale features into a unified sequence of image features. AIFI focuses on intra-scale information interaction: at each scale, features are independently processed through a transformation layer before being concatenated, thereby fully exploiting contextual correlations at the same resolution while avoiding cross-scale interference. CCFM, in contrast, performs cross-scale feature integration by hierarchically fusing low-level fine-grained spatial details with high-level semantic information, significantly improving the model’s ability to detect objects of varying sizes. Following the encoding process, a fixed number of candidate features are selected from the encoder’s output sequence using the IoU-aware query selection strategy to serve as the initial query vectors for the Transformer decoder. Collaborating with auxiliary prediction heads, the decoder iteratively refines these queries and ultimately outputs the corresponding target bounding boxes along with their confidence scores. To satisfy the dual demands of low computational cost and high real-time performance in traffic scene object detection, this study adopts the RT-DETR-L variant with fewer parameters as the baseline model. The network architecture of RT-DETR-L is shown in Figure 1.

The scale variation in traffic targets presents an extremely wide range, often resulting in prevalent issues such as high false positive and missed detection rates, as well as notable localization inaccuracies for small objects, in existing detection models. To effectively address these challenges, we propose Trans-DETR, a novel detection framework that simultaneously enhances scale robustness and localization precision through three core components: the Multi-Scale Attention Module (MSAM), the LiteSNet, and the Reparameterized Detection Head (RepHead). The overall architecture of Trans-DETR is illustrated in Figure 2.

### 3.1. Multi-Scale Attention Module (MSAM)

We propose the MSAM to enhance the feature extraction capability of the model encoder. This module improves multi-scale feature fusion through channel attention, employs multi-branch deep convolution for precise extraction of multi-scale features, and integrates spatial attention in an efficient manner. As illustrated in Figure 3, MSAM comprises a channel attention module and a spatial attention module. The channel attention module incorporates Multi-Scale Convolutional Attention (MSCA), which enables cross-scale feature fusion by adaptively recalibrating channel-wise weights. It is primarily composed of the following three components:

- Multi-Scale Convolution: This uses convolution kernels of different sizes (such as 7 × 1, 11 × 1, 21 × 1, etc.) to extract features. These convolution kernels of different sizes can capture information from different receptive fields, thereby enhancing the model’s ability to detect targets of different sizes. The multiple convolution kernels (d, 7 × 1, d, 11 × 1, etc.) in the figure extract spatial features from different scales, obtaining multi-scale information from local to global.
- Channel Mixing: Following the multi-scale convolution operations, features across all scales are fused through the channel mixing operation. This process integrates information from different channels using 1 × 1 convolution, thereby enhancing the interdependencies among features. By employing 1 × 1 convolution for channel mixing, the model can significantly reduce both the number of parameters and computational complexity.
- Convolutional Attention: The final component of the MSCA module is the convolutional attention mechanism, which leverages convolution operations to identify and emphasize key regions within the image. By applying spatially adaptive feature weighting, this mechanism enhances the representation of important feature regions while suppressing irrelevant background noise. This process effectively guides the model to prioritize critical information, thereby improving feature discrimination and overall detection performance.

The spatial attention module employs the Spatial Attention Module (SAM) from Convolutional Block Attention Module (CBAM) to identify critical spatial positions on the feature map following channel dimension reduction. The MSAM module receives encoder-end features as input and generates outputs that encode both global contextual information and precise spatial location details. If the input feature is $F\in R^{C\times H\times W\times D}$, where *C*, *H*, *W*, and *D* correspond to the number of channels, height, width, and depth of the feature map, respectively. MSAM successively computes the multi-scale learning attention map $M_{\mathrm{Mul}-\mathrm{scale}}\in R^{B\times C\times H\times W\times D}$ and the position information attention map $M_{\mathrm{Position}}\in R^{B\times 1\times H\times W\times D}$, which are used to weight the input feature map *F* to obtain the feature map $F^{\prime \prime }$, as shown in Equation (1).

(1) $$\begin{array}{cc} F^{\prime } & =M_{\mathrm{Mul}-\mathrm{scale}}\left(F\right)⊗F\\ F^{\prime \prime } & =M_{\mathrm{Position}}\left(F^{\prime }\right)⊗F^{\prime } \end{array}$$

Here, $F^{\prime }$ is an intermediate result, and ⊗ represents multiplication.

### 3.2. LiteSNet

The RT-DETR-L model utilizes the structurally redundant HGNet-v2 as its backbone network, which results in suboptimal detection efficiency. To address this limitation, we propose a lightweight backbone network termed LiteSNet. This network is built upon cascaded horizontal–vertical separable convolutions, which not only maintain the model’s multi-scale feature representation capability but also effectively reduce parameter redundancy and computational overhead.

StarNet integrates the star operation into its network architecture, which optimizes feature extraction through sparse computation and effectively reduces the complexity of the network [11]. The overall architecture of StarNet is illustrated in Figure 4. The star operation combines features from two linear transformations via element-wise multiplication, and its mathematical formulation is given as follows:

(2) $$\left(W_{1}^{T}X+B_{1}\right)*\left(W_{2}^{T}X+B_{2}\right)$$

In this formulation, *W* denotes the weight matrix of the linear layer, *B* denotes the bias term, and *X* represents the input, with * indicating the star operation. By defining $W_{1},W_{2},$$X\in R^{(d+1)\times 1}$, where *d* represents the number of input channels, the star operation can be reformulated as follows:

(3) $$\begin{array}{c} W_{1}^{T}X*W_{2}^{T}X=\left(\sum_{i=1}^{d+1}W_{1}^{i}X^{i}\right)*\left(\sum_{j=1}^{d+1}W_{2}^{j}X^{j}\right)\\ =\underset{\frac{\left(d+2)(d+1\right)}{2}}{\underset{︸}{\alpha_{\left(1,1\right)}X^{1}X^{1}+\cdots +\alpha_{\left(d+1,d+1\right)}X^{d+1}X^{d+1}}} \end{array}$$

Here, *i* and *j* are index channels, and α is the coefficient of each item. This operation significantly amplifies the feature dimension without incurring additional computational overhead.

StarNet is a four-stage hierarchical network architecture. Each stage performs downsampling via convolutional layers and employs StarBlock modules for feature extraction. StarNet substitutes traditional layer normalization with batch normalization (BN), positioning it after the depthwise convolution to enable efficient fusion during inference. Furthermore, an additional depthwise convolution is incorporated at the end of each StarBlock to enhance feature extraction performance. The channel expansion factor is set to 4 across the network design, with the network width progressively doubled at each stage. To minimize computational cost and accelerate inference, the activation function is changed from GELU to the more efficient ReLU6.

Each StarBlock in StarNet performs feature extraction using a 7 × 7 depthwise convolution, which results in a significant computational cost. As illustrated in Figure 5, the proposed backbone network LiteSNet addresses this issue by decomposing the large convolutional kernel into a two-stage process involving horizontal and vertical convolutions:

- First stage: 1 × k horizontal convolution (corresponding to ks = (1, 5), padding = (0, 2) and ks = (1, 3), padding = (0, 2));
- Second stage: k × 1 vertical convolution (corresponding to ks = (5, 1), padding = (2, 0) and ks = (3, 1), padding = (2, 0), along with other configurations).

To compensate for the receptive field reduction caused by depthwise convolution, LiteSNet introduces dilated convolution with a dilation rate of 2 after the 1 × k and k × 1 depthwise convolutions. This strategy expands the effective receptive field from k to 2k × 1 without introducing additional parameters, preserving a receptive field comparable to that of the 7 × 7 convolution in StarNet, while reducing computational cost to approximately 4% of the original ($2/7^{2}\approx 4\%$). For example, when k = 5, the receptive field reaches 9, closely matching the 7 × 7 convolution in StarNet (receptive field = 7). Within LiteSNet, the LSBlock applies a 1 × 1 convolution after DW-Conv to compress the channel dimension from 4d to d, followed by recalibration via fully connected layers (FC(d), FC(4d) in Figure 5), thereby enabling cross-channel information interaction. LiteSNet adopts StarNet’s star operation, performing element-wise multiplication between the recalibrated features and the corresponding input branch, and adding the result to the main branch to construct a residual connection.
**Algorithm 1** LSBlock: Lightweight Separable Conv Block with Dilated Aggregation**Require:** Input feature map $x\in R^{C\times H\times W}$; channel *C*; MLP ratio r=3 **Ensure:** Refined feature $y\in R^{C\times H\times W}$  1:{Stage 1: Decomposed depthwise convolutions}  2:$x_{1}\leftarrow {\mathrm{ConvBN}}_{1\times 5}\left(x\right)$  3:$x_{1}\leftarrow {\mathrm{ConvBN}}_{5\times 1}\left(x_{1}\right)$  4:$x_{1}\leftarrow {\mathrm{ConvBN}}_{1\times 3}^{d=2}\left(x_{1}\right)$  5:$x_{1}\leftarrow {\mathrm{ConvBN}}_{3\times 1}^{d=2}\left(x_{1}\right)$  6:{Stage 2: Star operation (channel expansion)}  7:$x_{2}\leftarrow {\mathrm{Conv}}_{1\times 1}\left(x_{1}\right)\in R^{rC\times H\times W}$  8:$x_{3}\leftarrow {\mathrm{Conv}}_{1\times 1}\left(x_{1}\right)\in R^{rC\times H\times W}$  9:$x_{4}\leftarrow \mathrm{ReLU}6\left(x_{2}\right)⊙x_{3}$10:{Stage 3: Projection + decomposed depthwise (no BN)}11:$x_{5}\leftarrow {\mathrm{ConvBN}}_{1\times 1}\left(x_{4}\right)\in R^{C\times H\times W}$12:$x_{5}\leftarrow {\mathrm{Conv}}_{1\times 5}\left(x_{5}\right)$13:$x_{5}\leftarrow {\mathrm{Conv}}_{5\times 1}\left(x_{5}\right)$14:$x_{5}\leftarrow {\mathrm{Conv}}_{1\times 3}^{d=2}\left(x_{5}\right)$15:$x_{5}\leftarrow {\mathrm{Conv}}_{3\times 1}^{d=2}\left(x_{5}\right)$16:{Stage 4: Residual connection}17:$y\leftarrow x+\mathrm{DropPath}(x_{5})$18:**return** y

### 3.3. Reparameterized Detection Head (RepHead)

The high-speed movement of vehicles can result in significant displacement of target positions within a short time frame, causing distant targets to occupy only a limited number of pixels in the image. This makes them highly susceptible to being missed or misclassified due to their small scale. To address this challenge, we propose a reparameterized detection head called RepHead, which incorporates the Cross Stage Partial Stage (CSPStage) structure. By integrating shallow spatial details with deep semantic features, the module effectively compensates for the limited detail representation capability of shallow features, thereby significantly enhancing the model’s performance in detecting small-scale targets.

The architecture of the RepHead is illustrated in Figure 6. The RepHead module incorporates five CSPStage modules to capture more node inputs, enabling the fusion of multi-scale image features from adjacent layers. This design enhances feature reuse and representation capabilities, thereby improving detection accuracy. The structure of the CSPStage module is presented in Figure 7. By integrating recursive operations and a reparameterization mechanism, the CSPStage module decouples the network architecture during training and inference. During the forward pass, the concatenated feature map is divided into two branches: one branch compresses the channel dimension using a 1 × 1 convolution, while the other is fed into the ELAN feature aggregation module, which consists of multiple 3 × 3 convolution operations. Finally, the features from both branches are concatenated and fused to generate the final output.
**Algorithm 2** RepHead: Reparameterized Detection Head**Require:** Multi-scale features $F_{s}$, s∈{3,4,5} from CCFM **Ensure:** Detection predictions P  1:{CSPStage: cross-stage partial fusion per scale}  2:**for** each scale *s* **do**  3:   $y_{1}\leftarrow {\mathrm{Conv}}_{1\times 1}\left(F_{s}\right)$ {preserve branch}  4:   $y_{2}\leftarrow {\mathrm{Conv}}_{1\times 1}\left(F_{s}\right)$ {process branch}  5:   $M\leftarrow [\;y_{1}\;]$  6:   **for** i=1 to *n* **do**  7:     $y_{2}\leftarrow \mathrm{RepConv}\left(y_{2}\right)+y_{2}$ {reparameterized residual}  8:     $y_{2}\leftarrow {\mathrm{Conv}}_{3\times 3}\left(y_{2}\right)$  9:     $M\leftarrow M\cup \{y_{2}\}$10:   **end for**11:   $F_{s}^{\mathrm{out}}\leftarrow {\mathrm{Conv}}_{1\times 1}\left(\mathrm{Concat}(M)\right)$12:**end for**13:$P\leftarrow \mathrm{Head}\left(\mathrm{Concat}\{F_{3}^{\mathrm{out}},F_{4}^{\mathrm{out}},F_{5}^{\mathrm{out}}\}\right)$14:**return** P


## 4. Experiments and Analysis

### 4.1. Network Training

The experiments were conducted on a platform running the Windows 10 operating system. The hardware configuration consists of an AMD Ryzen 7 9700X processor and an NVIDIA GeForce RTX 4070 Ti SUPER graphics card, equipped with 16 GB of video memory and 64 GB of system memory.

The Trans-DETR model was trained on the KITTI dataset, which comprises 15,000 images representing eight categories of urban road traffic objects, including Car, Van, Truck, Pedestrian, and Cyclist. In this study, the dataset was partitioned into a training set of 6981 images, a validation set of 500 images, and a test set of 7518 images to support model training and performance evaluation. The model training results are visualized in Figure 8. After 500 epochs of training, both the training loss and validation loss converge and stabilize, indicating effective learning and generalization.

### 4.2. Evaluation Index

The experimental results were analyzed using metrics such as mean Average Precision (mAP), Floating Point Operations (FLOPs), and Latency time to evaluate the performance of the model.

(4) $$\begin{array}{c} AP=\int_{0}^{1}PdR\\ mAP=\sum_{j}^{class}Class_{A}P_{j} \end{array}$$

AP denotes the detection accuracy for a single category, whereas mAP serves as a comprehensive performance metric calculated by averaging the AP values across all categories. A higher mAP value reflects improved overall detection accuracy. $mAP_{50}$ denotes the mean average precision (mAP) computed across all object categories at a fixed intersection over union (IoU) threshold of 0.5. This metric provides a relatively lenient evaluation standard and is commonly used for initial model performance assessment. In contrast, $mAP_{50\text{-}95}$ is calculated by averaging the average precision (AP) values across ten uniformly sampled IoU thresholds ranging from 0.5 to 0.95 (with a step size of 0.05), offering a more comprehensive and rigorous evaluation of detection accuracy.

FLOPs is an important metric for evaluating the computational cost of a model. If the input feature size is $H\times W\times C_{in}$, the convolution kernel size is *k*, then the output feature size is $H\times W\times C_{out}$, and the number of parameters in the convolution kernel is $k^{2}$.

(5) $$\begin{array}{c} FLOPs=H\times W\times C_{in}\times C_{out}\times k^{2} \end{array}$$

Latency time is defined as the duration required for a neural network to perform forward propagation on a single input image, excluding any post-processing stages.

(6) $$\begin{array}{c} Latency=\frac{\sum_{j=1}^{N}T_{j}}{N} \end{array}$$

Here, *N* represents the number of images, and $T_{j}$ indicates the time taken by the algorithm to process the *j*th image. Generally, a lower value indicates that the model can process a single image more rapidly, thereby demonstrating enhanced real-time performance.

### 4.3. Result Analysis and Discussion

Ablation study on the KITTI dataset quantify the individual contributions of MSAM, LiteSNet and RepHead (Table 1). The baseline RT-DETR-L achieves 87.8% $mAP_{50}$ and 54.2% $mAP_{50\text{-}95}$. The model has 125.1 MB parameters, 108.0 GFLOPs and 4.3 ms latency. MSAM raises $mAP_{50\text{-}95}$ by 2.8 percentage points to 57.0%, at the expense of only 2.9 MB extra parameters, 4.5 GFLOPs, and 1.2 ms latency, indicating that multi-scale convolutional attention strengthens cross-scale feature discrimination with marginal overhead. LiteSNet reduces parameters to 42.5 MB and GFLOPs to 29.4, cutting latency to 2.9 ms. Notably, $mAP_{50\text{-}95}$ rises by 4.2 percentage points to 58.4% despite an 82.6 MB parameter reduction, whereas $mAP_{50}$ drops by 2.8 percentage points. The concurrent improvement in localization robustness suggests that decomposed large-kernel separable convolutions with dilated receptive fields maintain representational capacity. RepHead improves $mAP_{50\text{-}95}$ by 9.4 percentage points to 63.6% and $mAP_{50}$ by 1.5 percentage points, with recall rising to 85.2% at the expense of precision. Concurrently, parameters fall to 109.4 MB and GFLOPs to 86.9. The cross-stage partial reparameterization structure therefore enables efficient multi-scale feature fusion with negligible latency overhead. The integrated Trans-DETR achieves 89.8% $mAP_{50}$ and 66.3% $mAP_{50\text{-}95}$. The model has 54.1 MB parameters, 47.8 GFLOPs and 4.6 ms latency. Precision and recall settle at 87.2% and 81.7%, respectively, indicating that the three modules jointly rebalance detection confidence and coverage to optimize the accuracy–efficiency trade-off. MSAM + LiteSNet retains MSAM’s accuracy gain while compressing parameters to 44.5 MB and latency to 3.8 ms, indicating that multi-scale attention persists on a lightweight backbone. MSAM + RepHead reaches 65.1% mAP50-95, within 1.2 percentage points of the full model, where RepHead corrects the localization residuals left by MSAM. LiteSNet + RepHead trades merely 1.1 percentage points of mAP50-95 for a halving of parameters (51.5 MB) and GFLOPs (41.3) over RepHead alone, confirming that the two modules address disjoint efficiency and accuracy axes. Notably, MSAM + RepHead alone recovers 90% of the full model’s mAP50-95 gain, whereas LiteSNet contributes primarily through a 59% parameter reduction rather than accuracy, clarifying the distinct contribution of each component to the overall improvement. The ablation confirms that the balance stems from complementarity rather than accumulation. LiteSNet is the only module that lowers mAP50 in isolation (−2.8 percentage points), and RepHead restores this loss in the combined model. The three modules target distinct axes. RepHead improves accuracy, LiteSNet reduces parameters and computation, and MSAM strengthens multi-scale discrimination at marginal overhead. Their costs therefore remain disjoint, and the integrated model advances accuracy and efficiency simultaneously rather than trading one for the other.

As presented in Table 2, the Trans-DETR model achieves an $mAP_{50}$ of 89.8% and an $mAP_{50\text{-}95}$ of 66.3% on the KITTI vehicle detection benchmark. Compared with RT-DETR-L, Trans-DETR improves $mAP_{50}$ by 2.0 percentage points and $mAP_{50\text{-}95}$ by 12.1 percentage points. Relative to RT-DETR-ResNet50, the proposed method achieves gains of 1.3 percentage points in $mAP_{50}$ and 2.3 percentage points in $mAP_{50\text{-}95}$. The model contains 54.1 MB of parameters and 47.8 GFLOPs of computational complexity, corresponding to 43% and 44% of those of RT-DETR-L, and 38% of the FLOPs of RT-DETR-ResNet50. These results indicate that Trans-DETR attains a favorable balance between detection accuracy and computational efficiency among the methods with complete parameter and FLOPs records in Table 2.

To further verify the generalization ability of Trans-DETR, we conducted comparative experiments on the PASCAL VOC2012 dataset (Table 3). Trans-DETR achieved an $mAP_{50}$ of 71.4%. While this is lower than YOLO-ESFM (83.1%) and YOLOv7 (82.3%), which are optimized for general object detection with substantially larger model capacities, Trans-DETR outperforms YOLOX-S (65.0%) and Faster RCNN (69.9%), and remains competitive with Retinanet (72.8%). Delving into the per-category breakdown, Trans-DETR achieves 85.2% on the Car category, surpassing Faster RCNN (83.74%), YOLOX-S (80.9%), YOLOX++S (81.9%), and WS-SAM (66%), while remaining comparable to YOLOv5s (85.9%). The Bus and Person categories are detected at 77% and 81.4%, respectively, outperforming YOLOX-S and WS-SAM by considerable margins. These per-category results substantiate that the proposed modules retain strong discriminative capability for traffic-relevant object categories even under cross-domain generalization, underscoring their practical value in intelligent transportation systems. The moderate performance gap on VOC2012 is primarily attributed to the domain-specific optimization of the proposed modules for traffic scene characteristics, which limits their full effectiveness in general scenes with diverse object categories. These results confirm that Trans-DETR delivers robust cross-scene generalization, and future work will focus on enhancing category adaptability through unsupervised domain adaptation techniques.

For a controlled comparison, both RT-DETR-L (baseline) and Trans-DETR (Ours) are evaluated on the same KITTI validation split (500 images, 6 classes: Car, Van, Truck, Pedestrian, Cyclist, Tram). The two differ only in network architecture, so any AP gap can be attributed to the proposed modules. Each ground-truth box is assigned to a scale according to the COCO area thresholds applied in the 640 × 640 input space (Small $<32^{2}$ px^2^, Medium $32^{2}$–$96^{2}$ px^2^, Large $>96^{2}$ px^2^), and AP is computed separately on each subset. Table 4 reports the scale-stratified results. Trans-DETR yields large gains on small objects (+3.4% AP_50_) and especially on medium objects (+14.7% AP_50_), showing that the proposed modifications concentrate their benefit on the scales where the baseline is weakest. On the large-object subset, Trans-DETR is lower than the baseline (Δ=−18.4% AP_50_). This result is consistent with the representational trade-off inherent in the proposed design: the MSAM and LiteSNet modules are introduced to strengthen multi-scale feature discrimination, and their contribution is concentrated at small and medium scales, where the baseline exhibits the greatest residual error. Consequently, the reweighting of the shared feature hierarchy toward these scales yields a marginal regression on large objects, which the baseline already localizes reliably (AP_50_ 65.5%). The overall mAP_50_ still improves from 87.8% to 89.8%, indicating that the substantial gains on the more challenging scales outweigh the localized degradation on large instances.

As summarized in Table 5, Trans-DETR achieves 45.14% mAP@0.5 and 24.69% mAP@0.5:0.95 on the BDD100K validation set, outperforming the RT-DETR-L baseline (43.27%/23.96%) by +1.87 and +0.73 percentage points, respectively. Since BDD100K is a challenging autonomous-driving benchmark whose scene distribution and object-scale diversity differ markedly from conventional detection datasets, the consistent improvement over a strong real-time baseline on this distinct domain indicates that the proposed detector generalizes well to complex, real-world traffic-scene perception.

In this study, we employed heatmaps to compare the feature attention regions of Trans-DETR and RT-DETR-L in the traffic object detection task. Figure 9 displays the heatmaps of selected images from the KITTI dataset for both algorithms. The analysis results indicate that Trans-DETR achieves more concentrated and precise attention on feature regions across various traffic scenarios. Notably, in scenes characterized by vehicle congestion and the presence of small objects, Trans-DETR exhibits superior focusing performance, which contributes to improved detection accuracy. Furthermore, the architectural components of Trans-DETR, including the MSAM module, LiteSNet, and RepHead, enhance its capability to detect small targets and improve the model’s robustness in complex visual environments. The heatmap-based comparative analysis provides intuitive evidence, further substantiating the effectiveness and superiority of Trans-DETR in traffic object detection.

To visually evaluate the detection performance of Trans-DETR, we conducted comparative visualization experiments by selecting representative images from the KITTI and PASCAL VOC2012 datasets, with results presented in Figure 10 and Figure 11. The experimental results demonstrate that Trans-DETR is capable of accurately localizing target regions in images and effectively detecting small-scale objects. Furthermore, in complex traffic scenarios characterized by varying object scales and challenging conditions such as occlusion and illumination variation, the model still achieves accurate detection of occluded vehicles and distant pedestrians that are typically difficult to detect.

## 5. Conclusions

This paper addresses the challenges of large-scale variations, strong background interference, and stringent real-time requirements in complex traffic scenarios by proposing Trans-DETR, a traffic target detection algorithm built upon the RT-DETR framework. The MSAM module enhances cross-scale feature representation, the lightweight backbone LiteSNet significantly reduces model parameters and computational cost, and RepHead integrates shallow spatial details with deep semantic information. Experimental results demonstrate that Trans-DETR achieves a favorable balance among detection accuracy, computational efficiency, and model compactness. This balance is not attained without cost, as accuracy, computational efficiency, and model compactness are intrinsically competing objectives in object detection. Trans-DETR reconciles them by design: the lightweight backbone reduces parameters and computation, the multi-scale attention improves representation at negligible overhead, and the redesigned head recovers accuracy through enriched feature fusion. Consequently, the model preserves detection accuracy while satisfying the strict efficiency and size requirements of real-time traffic perception, rather than sacrificing one objective for another.

While the proposed method achieves promising performance, several limitations remain. Performance under extreme weather conditions, including heavy rain, fog, and snow, remains to be systematically validated. Detection accuracy degrades for heavily occluded small targets in highly congested scenes. In addition, the current architecture, while lightweight relative to RT-DETR-L, has yet to be optimized for deployment on resource-constrained edge devices. Future research [44] will accordingly focus on leveraging unsupervised domain adaptation, self-supervised pre-training, and multi-modal perception techniques to enhance the model’s generalization performance and deployment flexibility under cross-domain, extreme weather, and low-lighting conditions, thereby providing more robust and reliable all-weather perception support for autonomous driving and intelligent transportation systems. 

## Figures and Tables

**Figure 1:**
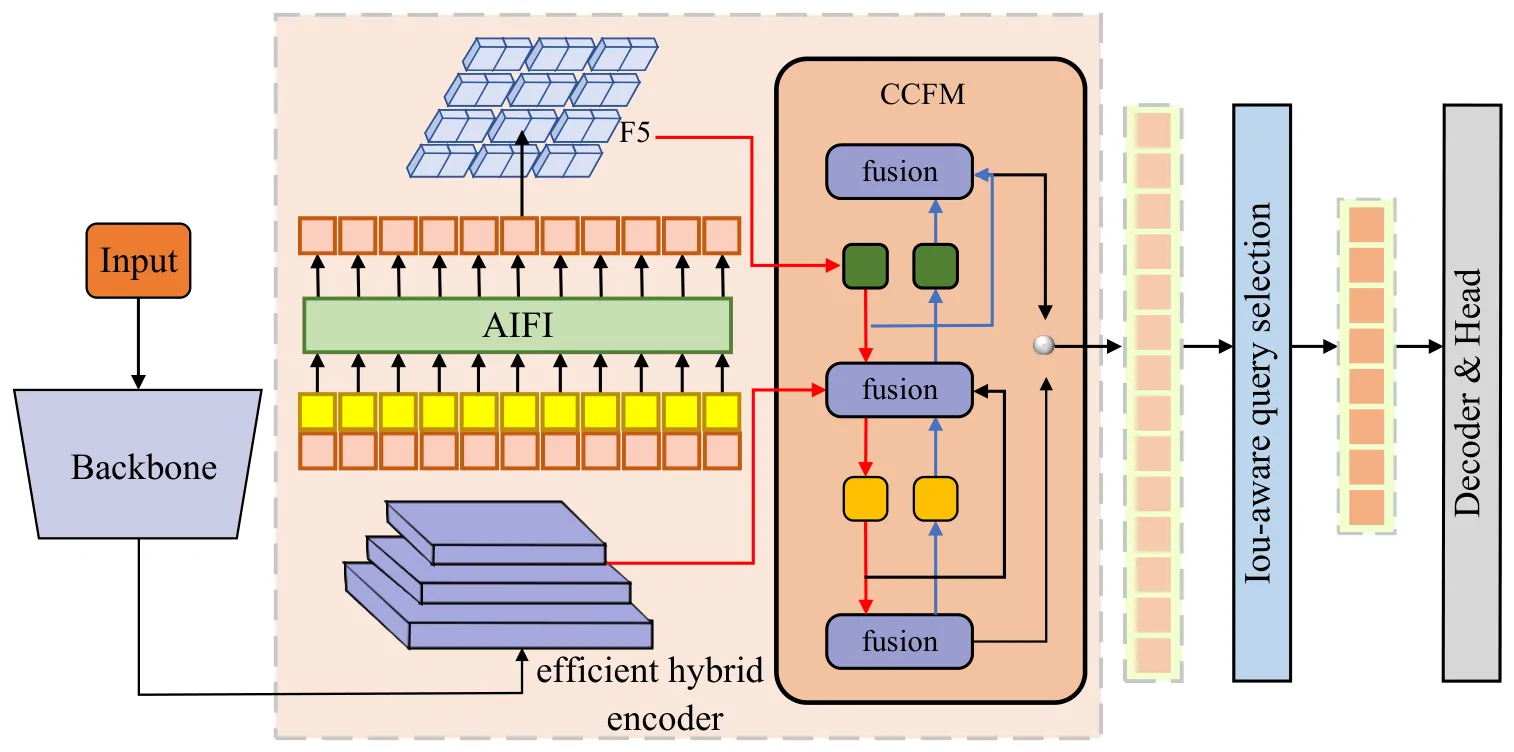
Network structure of RT-DETR-L.

**Figure 2:**
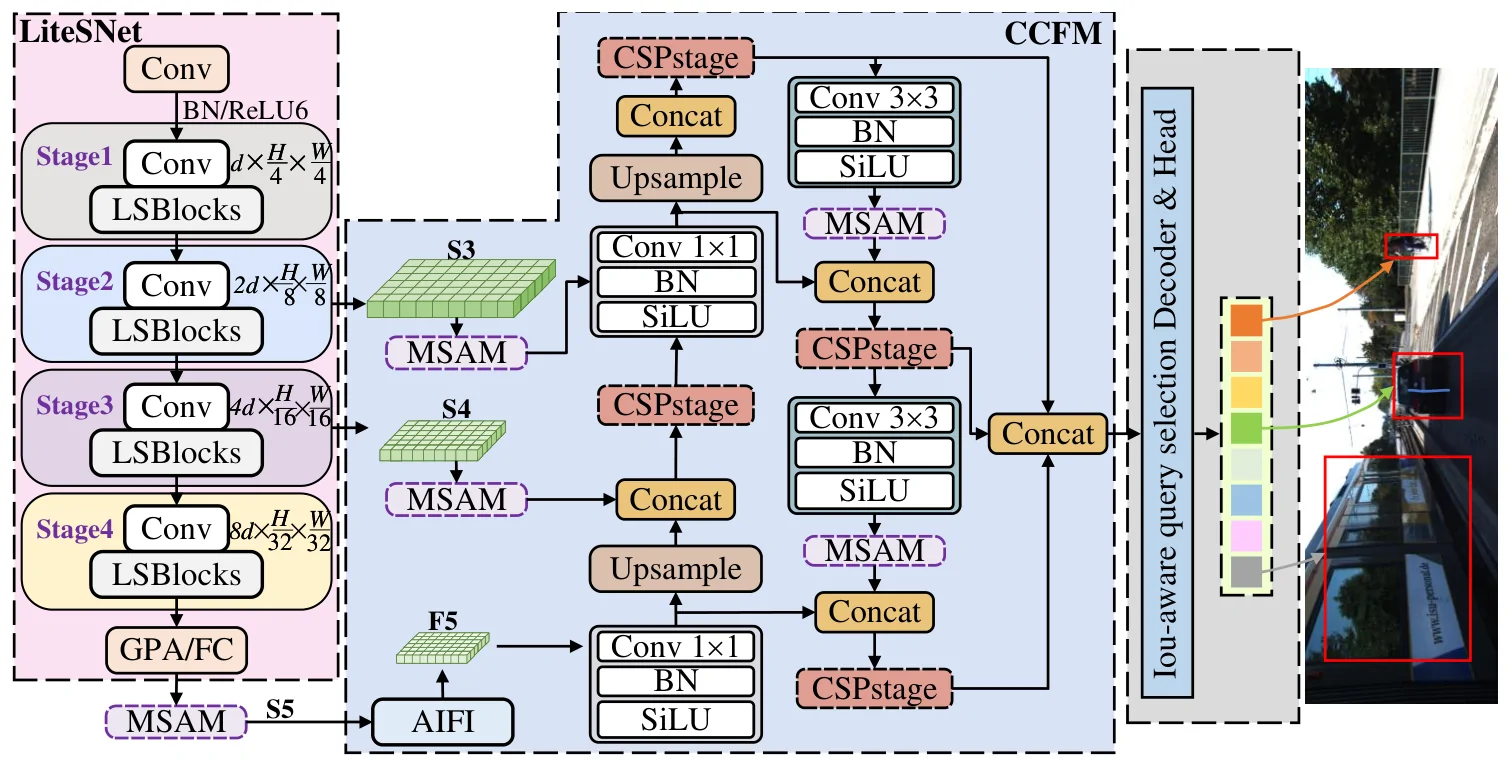
Trans-DETR network architecture.

**Figure 3:**
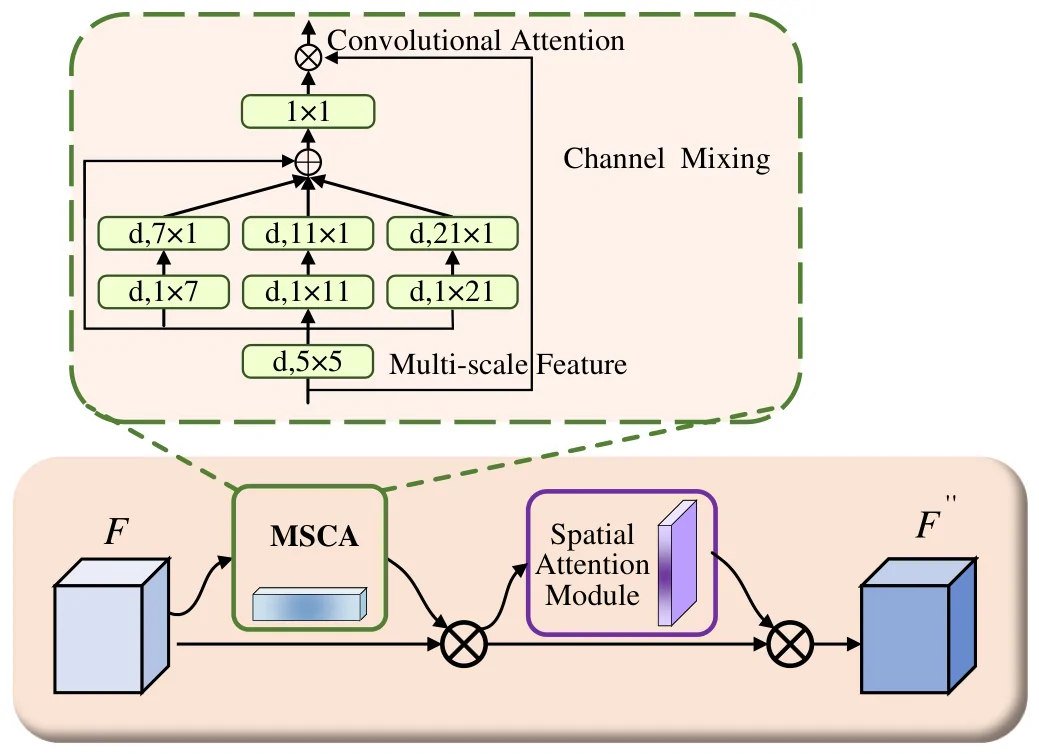
MSAM module structure.

**Figure 4:**
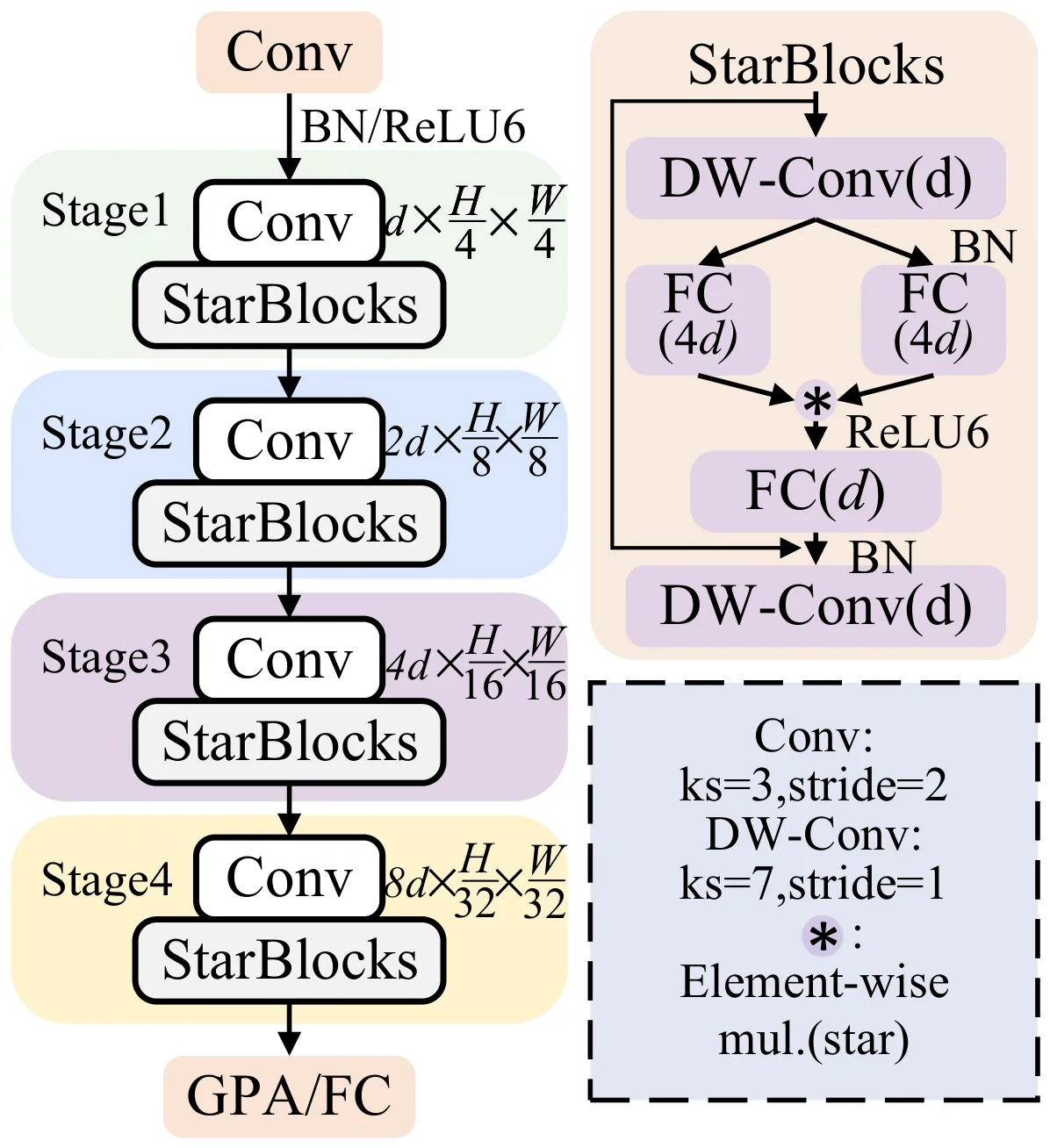
StarNet network.

**Figure 5:**
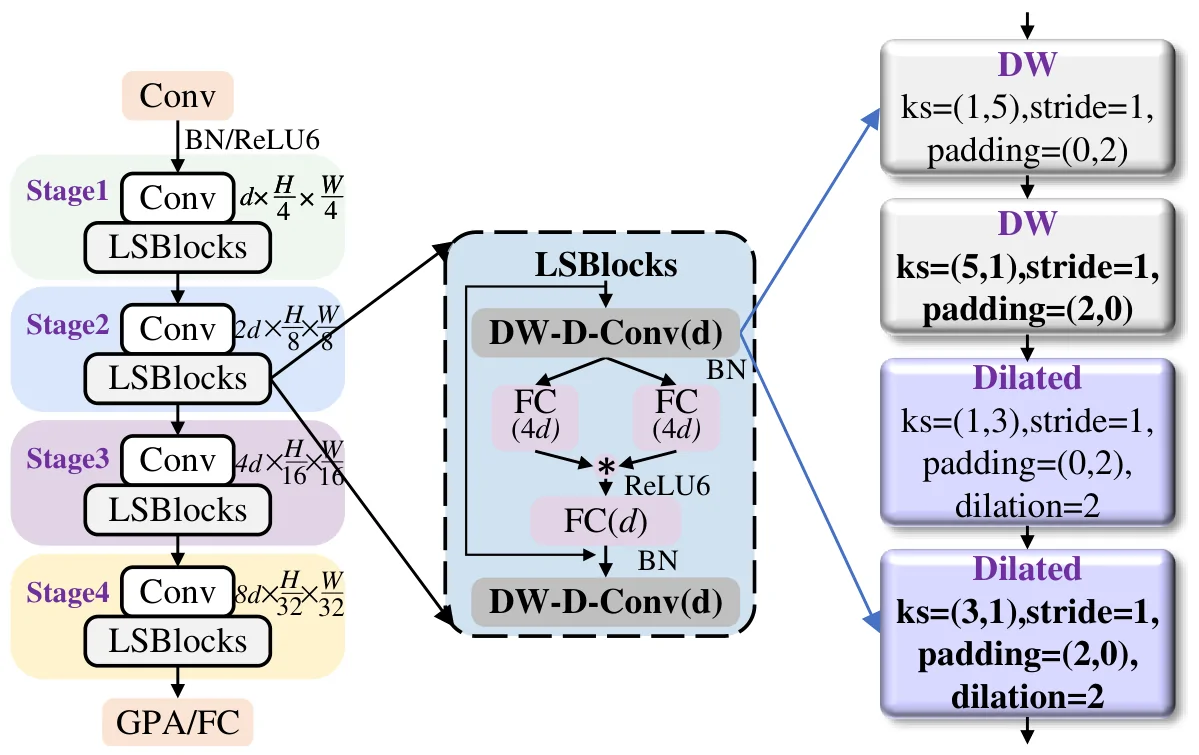
LiteSNet structure. LSBlock denotes the lightweight separable convolution block with dilated aggregation (detailed in Algorithm 1), and * denotes the star operation (element-wise multiplication).

**Figure 6:**
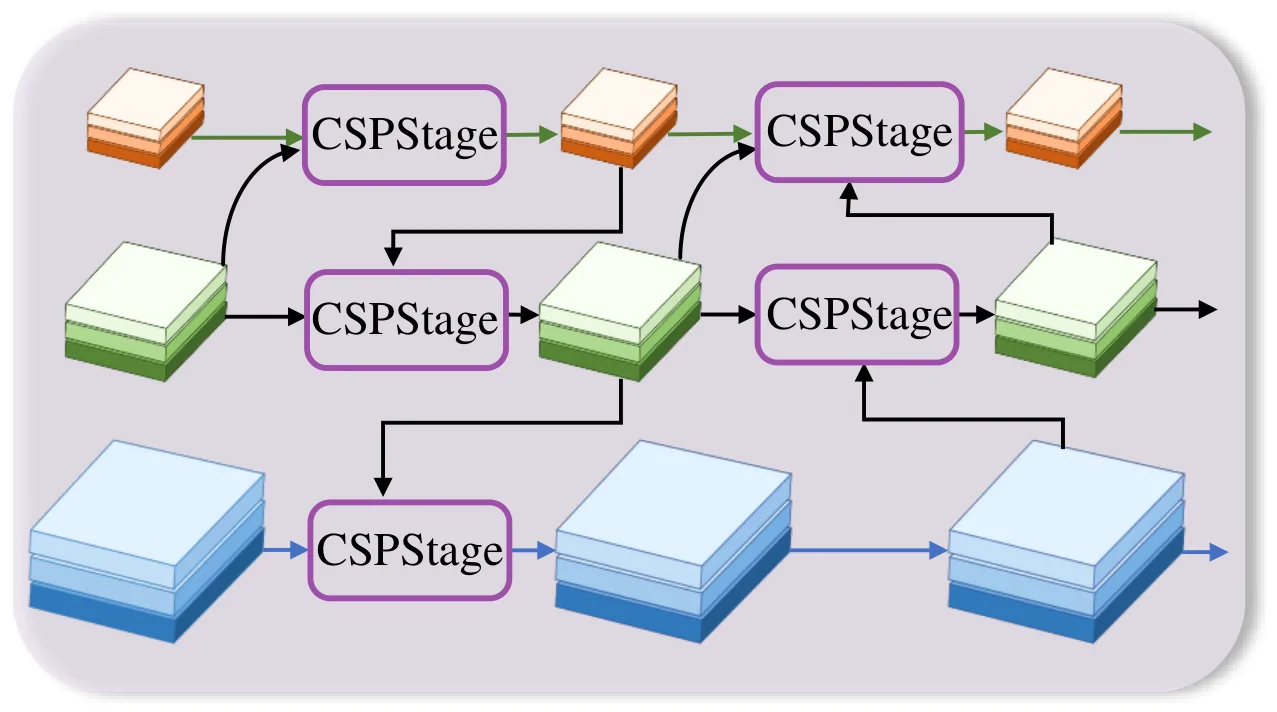
RepHead structure. CSPStage denotes the cross-stage partial fusion module with reparameterization (detailed in Algorithm 2).

**Figure 7:**
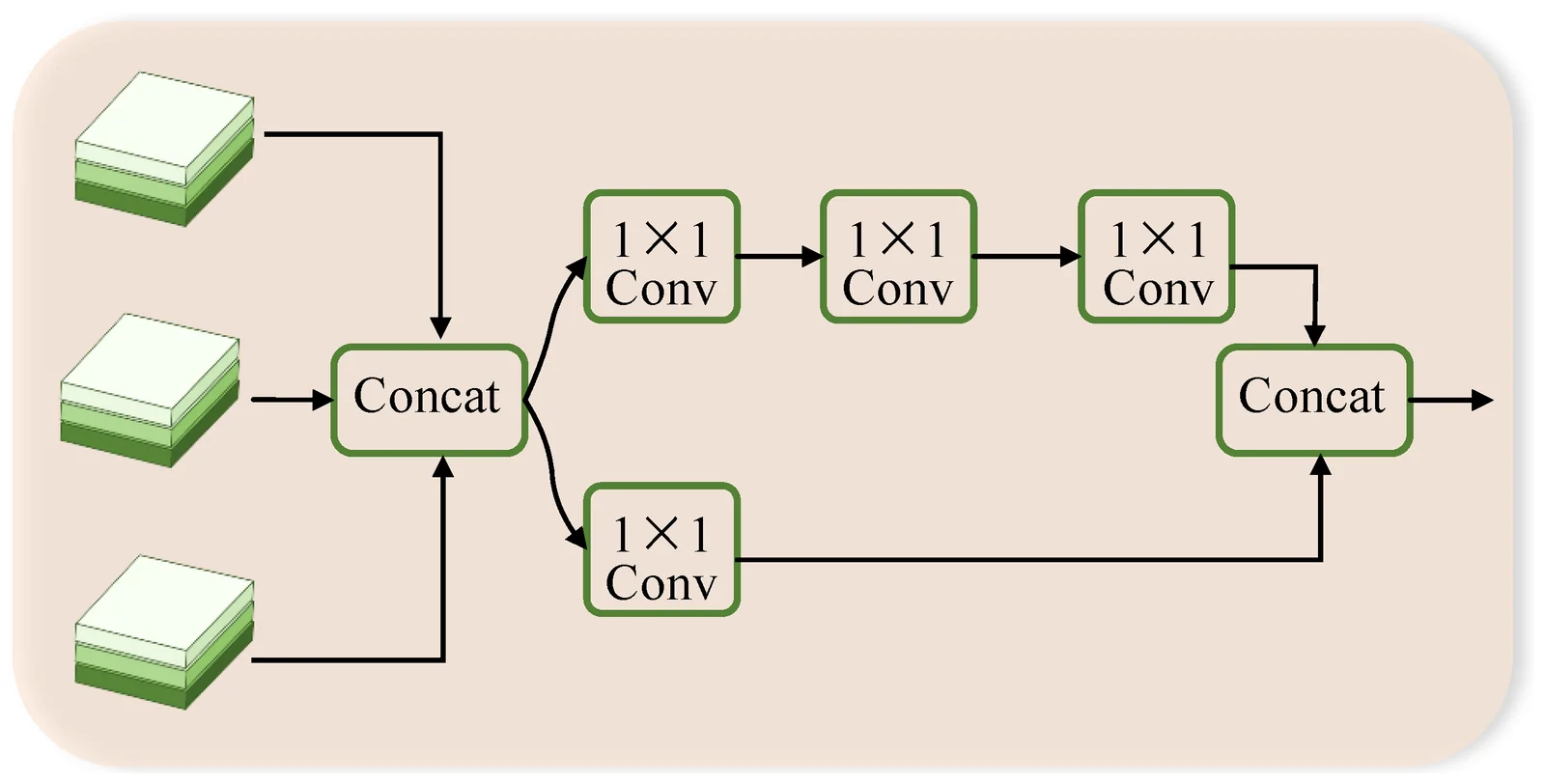
CSPStage module.

**Figure 8:**
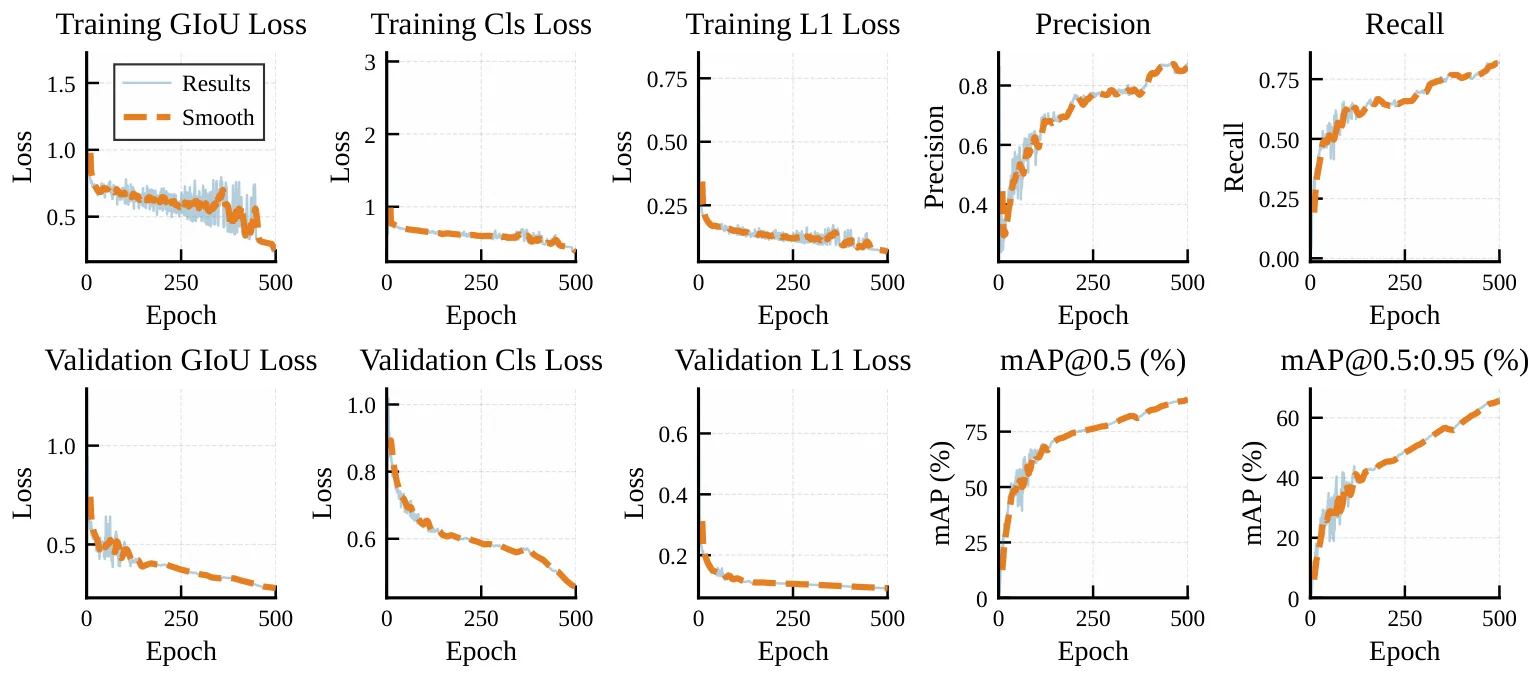
Model training results.

**Figure 9:**
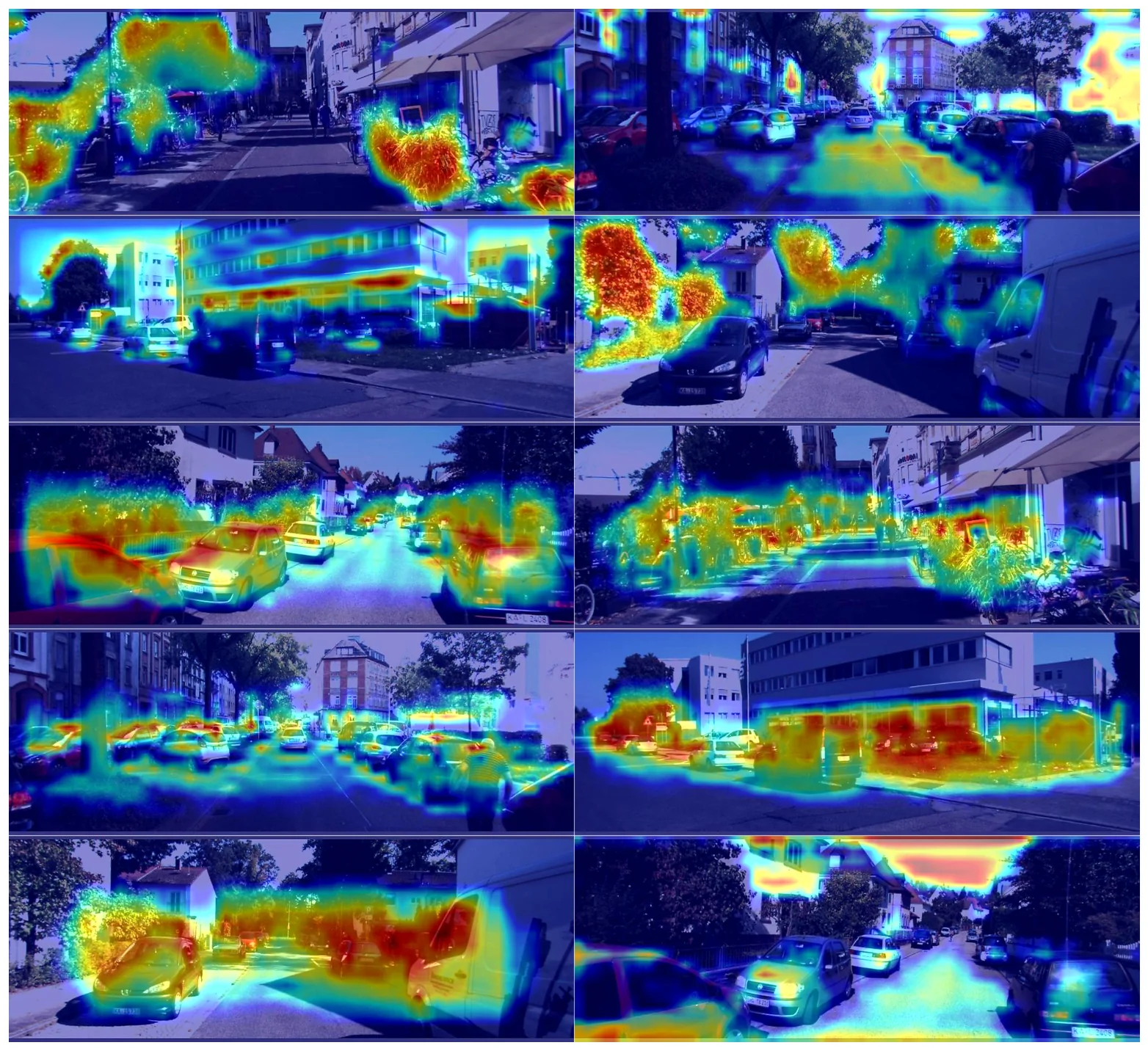
Heatmap comparison of detection algorithms.

**Figure 10:**
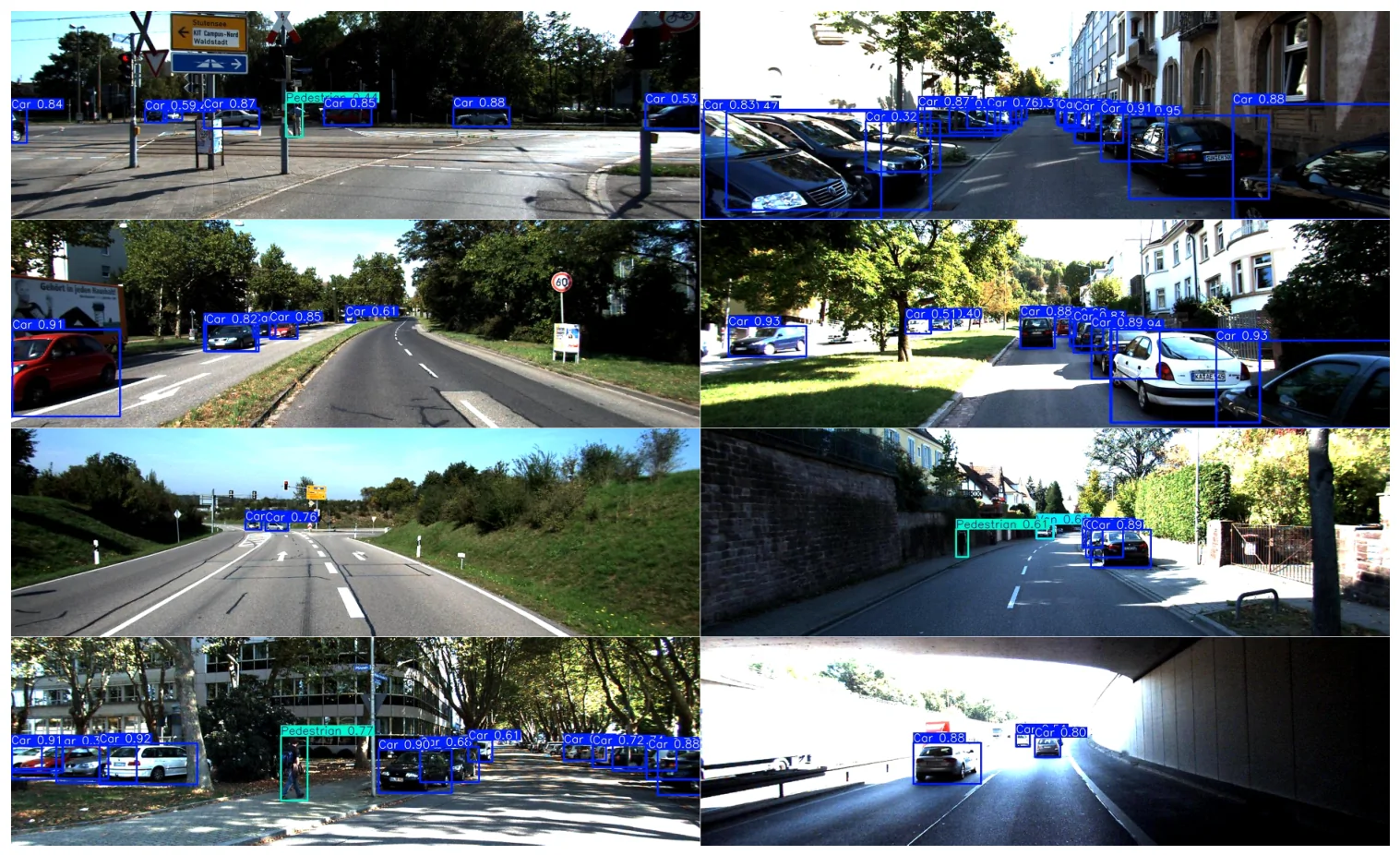
Visualization results on the KITTI dataset.

**Figure 11:**
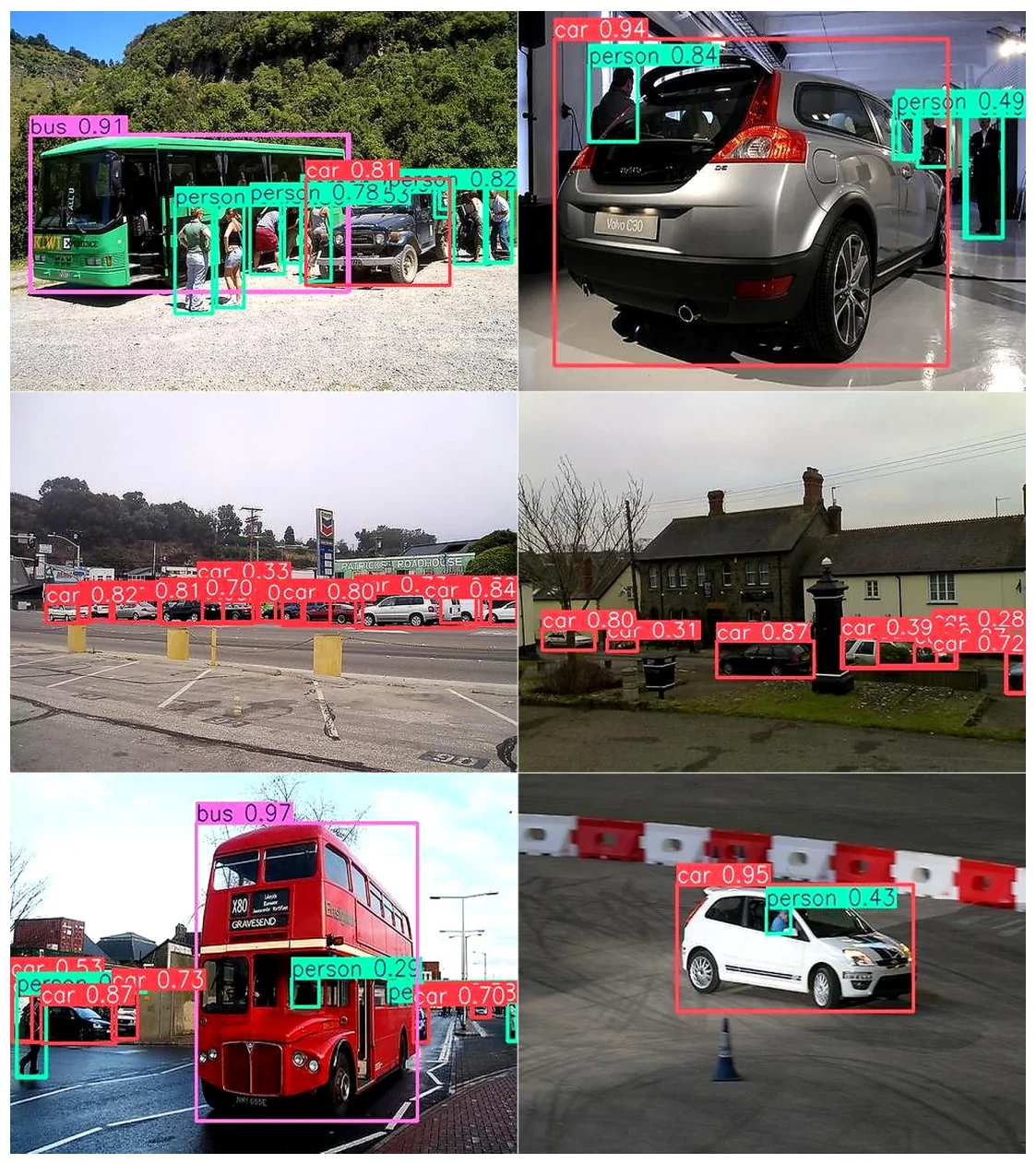
Visualization results on the VOC2012 dataset.

**Table 1:** Ablation study of the proposed modules.

Module			Accuracy				Efficiency		
MSAM	LiteSNet	RepHead	P/%	R/%	${\mathrm{mAP}}_{50}/\%$	${\mathrm{mAP}}_{50\text{-}95}/\%$	Params/MB	GFLOPs	Latency/ms
			87.1	79.2	87.8	54.2	125.1	108.0	4.3
✓			89.9	78.3	88.5	57.0	128.0	112.5	5.5
	✓		82.5	77.0	85.0	58.4	42.5	29.4	2.9
		✓	83.1	85.2	89.3	63.6	109.4	86.9	4.4
✓	✓		83.3	78.8	87.4	60.9	44.5	35.9	3.8
✓		✓	88.7	83	89.2	65.1	52.1	95.8	5.7
	✓	✓	87.7	81.8	87.5	62.5	51.5	41.3	4.1
✓	✓	✓	87.2	81.7	89.8 (+2.0)	66.3 (+12.1)	54.1	47.8	4.6

Note: ✓ indicates that the corresponding module is included in the model.

**Table 2:** Performance comparison with other advanced algorithms on the KITTI benchmark dataset.

Method	${\mathrm{mAP}}_{50}/\%$	${\mathrm{mAP}}_{50\text{-}95}/\%$	Params/MB	FLOPs/G
YOLO-ELWNet [31]	85.08	-	23.87	20.01
CenterNet [32]	73.92	-	32.67	70.22
YOLOX-M [33]	76.28	-	25.33	73.98
RT-Vehicle [10]	86.8	64.9	16.8	57
CEEP-Det [34]	74.9	-	288.5	-
SES-YOLOv8n [35]	92.7	69.2	-	-
YOLOv11 [36]	85.9	-	2.46	-
CTS-YOLO [9]	82.5	56.8	2.15	9.3
RT-DETR-ResNet50 [30]	88.5	64	288.5	125.6
RT-DETR-L [30]	87.8	54.2	125.1	108
Ours	89.8	66.3	54.1	47.8

**Table 3:** Comparison with advanced algorithms on the VOC2012 dataset.

Method	${\mathrm{mAP}}_{50}/\%$	Car/%	Bus/%	Bicycle/%	Person/%
Faster RCNN [37]	69.9	83.74	83.78	81.08	80.61
Retinanet [38]	72.8	86.94	84.4	85.26	81.77
Efficientdet [39]	74.23	84.44	82.79	80.86	82.35
YOLOv5s	75.4	85.9	80.9	83.4	83.6
YOLOX-S [33]	65	80.9	61.7	76.1	81.2
YOLOX++S [40]	74.3	81.9	74.6	89	84.1
YOLO-ESFM [41]	83.1	91	85.7	88.1	88.8
YOLOv7 [42]	82.3	90.8	86.7	88.8	88.2
WS-SAM [43]	75.8	66	68.8	76.9	20.4
Ours	71.4	85.2	77	76.5	81.4

**Table 4:** Object-scale stratified AP on KITTI (COCO area definition: Small <32^2^ px^2^, Medium 32^2^–96^2^ px^2^, Large >96^2^ px^2^). Δ is the improvement of Trans-DETR over RT-DETR-L.

Method	AP_50_/%			AP_50-95_/%		
	Small	Medium	Large	Small	Medium	Large
RT-DETR-L (baseline)	12.1	48.6	65.5	7.4	33.8	51.7
Trans-DETR (Ours)	15.4	63.3	47.1	10.2	46.9	37.0
Δ (Ours − Base)	+3.4	+14.7	−18.4	+2.8	+13.1	−14.7

**Table 5:** Comparison of the proposed Trans-DETR and the RT-DETR-L on the BDD100K validation set.

Method	mAP@0.5 (%)	mAP@0.5:0.95 (%)
RT-DETR-L	43.27	23.96
Trans-DETR	45.14	24.69

## Data Availability

The data that support the findings of this study are available from the corresponding author upon reasonable request.
